# Evaluating the impact of high risk foot training on undergraduate podiatry students

**DOI:** 10.1186/1757-1146-6-S1-O7

**Published:** 2013-05-31

**Authors:** Damien Clark, Lloyd Reed, Ewan M Kinnear, Peter A Lazzarini

**Affiliations:** 1 School of Clinical Sciences, Queensland University of Technology, Kelvin Grove, QLD, 4059, Australia; 2 Department of Podiatry, Metro North Hospital and Health Service, Queensland Health, Chermside, QLD, 4032, Australia; 3 Allied Health Research Collaborative, Metro North Hospital and Health Service, Queensland Health, Chermside, QLD, 4032, Australia; 4 Institute of Health and Biomedical Innovation, Queensland University of Technology, Kelvin Grove, QLD, 4059, Australia

## Background

Diabetes is the leading cause of high risk foot (HRF) complications, admissions and lower limb amputation. Best practice training of podiatrists is known to have a beneficial impact on such outcomes; however, there has been a paucity of studies into undergraduate diabetes podiatry training. The primary aim of this paper was to investigate the changes in final year podiatry students’ confidence, knowledge and clinical practice in the management of HRF complications.

## Methods

This was a prospective longitudinal study of final year podiatry students (n=25) at the Queensland University of Technology. All participants throughout 2011 undertook an intervention of a series of “hands on” HRF workshops, on-campus clinics and external clinical rotations. Outcome measures included customised confidence and knowledge surveys in HRF management across four time points. A timed simulated case scenario was used to evaluate changes in clinical practice at two time points. Friedman and Wilcoxon Signed Rank Tests were used to calculate differences between time points

## Results

Overall improvements between the first and last time points were demonstrated in 20/21 confidence items (p<0.001), 12/27 clinical practice items (p<0.05) and 3/12 knowledge items (p<0.001). Although 8/12 knowledge items recorded high baseline scores of over 80%.

## Conclusions

Overall, it appears student confidence and clinical practice improved with the introduction of designated HRF activities, whilst knowledge remained high. This suggests “hands on” practice and not didactic lectures improve students’ clinical confidence and practice. Results from the 2012 student cohort will also be presented at this conference.

